# Digital Dentistry as a New Culture of Learning and Practice

**DOI:** 10.21142/2523-2754-1304-2025-258

**Published:** 2025-11-08

**Authors:** César-Augusto Padilla-Avalos

**Affiliations:** 1 Research Professor, Research and Innovation Laboratory in Digital Dentistry, School of Dentistry, Universidad Científica del Sur. Lima, Peru. cpadillaav@cientifica.edu.pe Universidad Científica del Sur Research Professor, Research and Innovation Laboratory in Digital Dentistry School of Dentistry Universidad Científica del Sur Lima Peru cpadillaav@cientifica.edu.pe

Dentistry has experienced a profound digital transformation in recent years. Each year brings new technological advances, and keeping pace with them can be a demanding task. Three-dimensional workflows, digital scanners, and CAD/CAM (Computer-Aided design and manufacturing) systems are now part of daily clinical reality. However, the true challenge is not only to adopt new tools but to educate professionals capable of critical thinking and sound decision-making. Diagnosis, planning, and daily practice have also evolved. Today, digital systems play a central role in education because they allow students to practice procedures before treating real patients ^1^. Reports from the FDI World Dental Federation and the World Health Organization highlight that digital transformation is more than a technical change; it represents a new way of understanding access and equity in modern dentistry ^2^^,^^3^.

Developing digital competence is not limited to mastering software or handling equipment. It requires understanding the purpose, timing, and reasoning behind each technological resource ^4^. Digital tools can support clinicians, yet they will never substitute human judgment. Clinical reasoning continues to be the foundation of dental care. For that reason, education should promote a balanced integration of digital innovation and professional judgment. This is how technology has become a tool that enhances care rather than replacing its human dimension.

Contemporary dentistry currently stands on a hybrid stage. Traditional techniques remain valuable and reliable, but digital approaches are steadily gaining ground ^5^. When both are combined intelligently, clinical procedures become more efficient, predictable, and environmentally sustainable. This sustainability arises from the reduced use of physical materials and the digitization of clinical processes. For example, intraoral scanners replace conventional impressions, and digital models substitute plaster casts, generating less non-recyclable waste. Similarly, the shift from conventional radiography to digital imaging eliminates the need for chemical developing solutions, which are harmful to the environment, by allowing the capture of virtual data instead. Although these changes may appear small, they significantly contribute to environmentally responsible dentistry. Digital practice not only enhances efficiency but also aligns with global sustainability goals by minimizing waste and promoting ecological awareness. Another advantage is the ability to synchronize digital information among different specialties, improving diagnosis and treatment planning.

Digital workflows have improved every stage of clinical care. Dentists can now obtain accurate three-dimensional data from facial structures, dentition, and soft tissues through intraoral scanning, as well as bone anatomy using computed tomography. These data enable analysis, virtual planning, and design. The process concludes with the fabrication of dental restorations, which may be produced by printing (additive manufacturing) or milling (subtractive manufacturing). A particularly relevant development is the integration of artificial intelligence (AI) into software and clinical devices ^6^. AI assists in data analysis and prediction of results, but it does not replace the dentist’s reasoning. Ultimately, decision-making must remain the clinician’s responsibility.

In today’s context, patients expect more than treatment outcomes; they seek experiences that reflect comfort, efficiency, and innovation. Many can now visualize their smile before starting therapy, which transforms their expectations. This trend requires educators to prepare dentists who can manage digital tools responsibly and confidently ^7^. Digital simulations, smile design, and visual presentations foster communication and trust between patients and clinicians. These strategies are closely linked to patient-reported outcome measures (PROMs), which express how individuals perceive their treatments. Dentistry is gradually becoming more focused on the patient’s experience, a transition that defines its present evolution.

Dental education is undergoing a similar shift. Virtual three-dimensional environments encourage students to develop technological and analytical abilities, preparing them to face the dynamics of a competitive professional world. The dentist of the twenty-first century is not only a clinician but also a data interpreter, designer, and ethical decision-maker. The essence of transformation lies not in technology itself but in the way, professionals think and care for their patients.

Ultimately, digital transformation extends far beyond the acquisition of software or equipment; it represents a new culture of learning and clinical practice. Technology contributes to progress, but its use must always be accompanied by ethical responsibility. Digital training is most effective when it connects directly to real clinical objectives and outcomes. The best results are achieved when technology complements, rather than replaces, professional reasoning. Digital dentistry must remain human, evidence-based, and adaptable to future innovation. The true goal is to employ these tools to provide safer, more predictable, and patient-centered care. Behind every digital file or 3D model, there is a real person with emotions, expectations, and trust. In summary, digital transformation does not replace traditional dentistry. It represents its evolution toward a smarter, more compassionate, and sustainable future.

## References

[1] Yamakami SA, Nagai M, Chutinan S, Ohyama H (2022). 3D digital technology as an alternative educational tool in preclinical dentistry. Eur J Dent Educ.

[2] World Dental Federation (2021). Vision 2030: Delivering Optimal Oral Health for All.

[3] World Health Organization (2021). Global Strategy on Digital Health 2020-2025.

[4] Beltes C, Delantoni A, Orhan K, Delantoni A, Orhan K (2024). Digital Dentistry.

[5] da Silva RLB, Kim JH, Markarian RA, Falacho R, Cortes DN, Costa AJM (2022). Introduction to digital dentistry. In: Cortes ARG, editor. Digital Dentistry.

[6] Saghiri MA, Vakhnovetsky J, Nadershahi N (2022). Scoping review of artificial intelligence and immersive digital tools in dental education. J Dent Educ.

[7] Sheba M, Comnick C, Elkerdani T, Ashida S, Zeng E, Marchini L (2021). Students' perceptions and attitudes about digital dental technology are associated with their intention to use it. J Dent Educ.

